# "Metabolic staging" after major trauma - a guide for clinical decision making?

**DOI:** 10.1186/1757-7241-18-34

**Published:** 2010-06-17

**Authors:** Philip F Stahel, Michael A Flierl, Ernest E Moore

**Affiliations:** 1 Department of Orthopaedic Surgery Denver Health Medical Center University of Colorado School of Medicine 777 Bannock Street Denver, CO 80204 USA; 2 Department of Surgery Denver Health Medical Center University of Colorado School of Medicine 777 Bannock Street Denver, CO 80204 USA

## Abstract

Metabolic changes after major trauma have a complex underlying pathophysiology. The early posttraumatic stress response is associated with a state of hyperinflammation, with increased oxygen consumption and energy expenditure. This hypercatabolic state must be recognized early and mandates an early nutritional management strategy. A proactive concept of early enteral "immunonutrition" in severely injured patients, is aimed at counterbalancing the negative aspects of hyperinflammation and hypercatabolism in order to reduce the risk of late complications, including infections and posttraumatic organ failure. Recently, the concept of "metabolic staging" has been advocated, which takes into account the distinct inflammatory phases and metabolic phenotypes after major trauma, including the "ischemia/reperfusion phenotype", the "leukocytic phenotype", and the "angiogenic phenotype". The potential clinical impact of metabolic staging, and of an appropriately adapted "metabolic control" and nutritional support, remains to be determined.

## Commentary

In a recent article published in the *Journal*, Aller and colleagues propose a modern perspective on the metabolic events associated with the inflammatory response to major trauma, which should guide therapeutic strategies [1]. The authors classified the metabolic changes after injury into three distinct phenotypes: (1) the "ischemia/reperfusion phenotype"; (2) the "leukocytic phenotype"; and (3) the "angiogenic phenotype". Using this innovative classification concept, the authors explain the metabolic alterations in association with the "classical" progression of posttraumatic inflammation. The first ("ischemia/reperfusion") phenotype represents the immediate, nervous system-related alteration in response to injury, in which neuronal and humoral responses and edema formation predominate. This phase is characterized by regulating the metabolic supply to cells via the least elaborate mechanism: diffusion. The second ("leukocytic") phenotype, is characterized as the intermediate (or "immune") phase of the metabolic response to trauma. This phase is characterized by leukocytic and bacterial infiltration of previously damaged tissues, which occurs in an edematous, oxygen-poor environment. The resulting post-shock hypercatabolism and hypermetabolism is related to a hyperdynamic response with increased body temperature, increased oxygen consumption, glycogenolysis, lipolysis, proteolysis and futile substrate cycling. The third ("angiogenic") phenotype is defined as the late (or "endocrine") phase of systemic response to injury. This phase is characterized by a return of oxidative metabolism, favoring angiogenesis in damaged tissues and organs. This process creates a capillary bed that facilitates tissue repair and regeneration.

In 1942, Cuthbertson was the first to describe distinct phases of the metabolic changes which occur after major trauma [2,3]. He characterized the *"ebb" *and the *"flow" *phases of posttraumatic metabolic alterations. The "*ebb" *phase is associated with a decline in body temperature and oxygen consumption, presumably aimed at reducing posttraumatic energy depletion. The brief duration of this phase limits its clinical relevance. In contrast, the *"flow" *phase occurs after resuscitation from a state of shock, which leads to an increased metabolic turnover, activation of the innate immune system and induction of the hepatic acute-phase response. This hypercatabolic condition leads to a significantly increased oxygen consumption and energy expenditure. The state of hypercatabolism has been associated with severe complications after major trauma, related to hyperglycemia, hypoproteinemia, and immunosuppression. The presence and significance of these metabolic alterations must be recognized and managed early in multiply injured patients [4,5]. The catabolic state requires an adjusted energetic balance with early protein substitution and hypercaloric nutrition. Early enteral nutrition has been advocated as the concept of choice for nutrition of polytraumatized and severely ill patients. In this regard, prospective randomized controlled trials in the 1980s have clearly demonstrated the positive effect of an early full enteral nutrition with a decreased posttraumatic infection rate, a shorter duration of hospital stay, and an improved overall outcome [6,7]. The concept of "immunonutrition" is exemplified by the enteral supplementation of glutamine, an essential amino acid which exerts metabolic benefits beyond its nutritional value by mediating immunological effects, such as induction of neutrophil phagocytic activity and oxidative burst [8]. In addition, glutamine is a precursor to the reducing agent glutathione and thus contributes to antioxidant effects and cellular protection from ischemia/reperfusion-mediated injury [9]. A prospective, randomized, double-blind controlled clinical trial demonstrated that glutamine supplementation reduces the incidence of multiple organ failure and death attributed to infections in critically ill patients [10]. In addition to glutamine, Ω- fatty acids have become an important nutritional supplementation for severely injured patients [11]. These long-chain polyunsaturated fatty acids derived from fish oil were shown to have potent anti-inflammatory properties, related to the attenuation of arachidonic acid-derived metabolites like thromboxane A _2 _and leukotriene B _4_, inhibition of leukocyte activation and chemotaxis, and attenuation of pro-inflammatory gene expression levels. Other nutritional supplements that promote anabolism in trauma patients include phospholipids, leptins, and anabolic hormones, such as thyroid hormones, growth hormone, and insulin. For example, growth hormone substitution has been shown to promote protein anabolism in severely injured patients.

**Figure 1:**
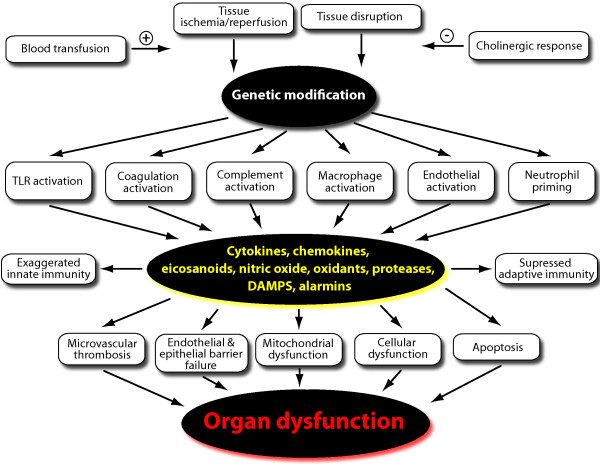
**Simplified schematic representing the current understanding of the pathophysiological reactions to major trauma, which lead to secondary remote organ dysfunction**. (Abbreviations: TLR, Toll-like receptor; DAMPs, damage-associated molecular patterns.)

The proposed metabolic classification in the paper by Aller and co-workers [1] is intuitively attractive, but is currently quite limited in application. The metabolic response to injury is complex and fundamentally driven by the combination of the primary events of tissue ischemia/reperfusion and tissue disruption. The response is further modified by innate gene expression and genetic polymorphisms, and aggravated by secondary events such as blood transfusions, delayed operative procedures, and infection [12,13]. These events provoke initiation of the cellular immune system (monocytes/macrophages, neutrophils, and endothelium), upregulation of Toll-like receptors (TLRs), activation of complement and coagulation cascades [12]. These immunological changes ultimately result in the notable release of a multitude of mediators including cytokines, chemokines, eicosanoids, oxidants, proteases, nitric oxide, alanine, and damage-associated molecular patterns (DAMPs)[12]. The released mediators are ultimately responsible for the metabolic response to injury as well as microvascular thrombosis, mitochondrial dysfunction, cellular necrosis and apoptosis, and ultimately secondary remote organ dysfunction (Figure 1). In fact, our therapeutic strategies to meet the metabolic needs of the injured patient have not gone much beyond the classic description of the *"ebb" *and *"flow" *phase proposal by Cuthbertson in 1942 [2,3]. As outlined above, the *"ebb" *phase, corresponding to the proposed "ischemia/reperfusion phenotype", is relatively brief, spanning < 12 hrs in most severely injured patients, with some extreme cases up to 24 hrs. During the resuscitation phase, there has been some evidence to initiate early β-blockade and antioxidant therapy, and even intestinal intraluminal glutamine administration, a concept which remains controversial [14]. As noted above, the brief duration of the initial phenotype phase limits its clinical relevance. The proposed "leukocyte phase" is where the focus on metabolic support has had the greatest impact on patient outcome. This period would physiologically correspond to Cuthbertson's *"flow" *phase where there is sustained hypermetabolism for at least 7 days, and in many severely injured patients for up to 3 weeks and longer [4,5]. During this initial period there is increased oxygen consumption, insulin resistance, and protein catabolism. Modest hyperglycemia is common due to increased hepatic glucose production and peripheral insulin resistance in skeletal muscle [15]. Changes in lipid metabolism include increased lipolysis, fatty acid recycling, hypertriglyceridemia, and hepatic steatosis [16]. The postinjury hypermetabolism is further characterized by increased skeletal and visceral muscle catabolism and negative nitrogen balance, leading to depletion of lean body mass, a syndrome which has been referred to as "autocannibalism" [17]. Glutamine released from muscle becomes the preferred energy substrate for enterocytes and immune cells, and is used to synthesize the antioxidant glutathione [18]. Hepatic protein synthesis is prioritized to generate acute phase proteins, such as C-reactive protein, at the expense of constitutive proteins, such as albumin and other carrier proteins [19]. Thus, appropriate nutritional therapy is integral in the management of severely injured patients that includes early enteral feeding, high protein administration, selective immunomodulation with diet enriched in glutamine and Ω- fatty acids [4,5,7]. Providing appropriate nutritional support becomes more challenging in patients who develop organ dysfunction as a result of their injuries and profound shock [20].

The authors propose a third, "angiogenic phenotype" [1]. But, again, the focus on angiogenesis may be somewhat myopic. On one hand, discerning the angiogenic phase from the leukocyte will be difficult as the two processes overlap. On the other hand, the nutritional needs are largely dictated by the metabolic state described in the leukocyte phase.

Based on these distinct pathophysiological phases of posttraumatic metabolic alterations, the authors deduce the need for a "metabolic staging" after severe trauma [1]. This implies the adjustment of the nutritional needs, which should be adjusted in a staged fashion to the different metabolic phases after major trauma. The authors conclude that a better understanding of these pathophysiological events may provide the treating clinician with novel and innovative therapeutic approaches, which include providing the most appropriate metabolic support dependent on the predominant phase and phenotype of metabolic alterations. The potential clinical impact and the feasibility of a, likely over-simplified, concept of "metabolic staging" in the guidance and decision making for the nutritional support of severely injured patients remains elusive.

## Competing interests

The authors declare that they have no competing interests.
